# Comparative Analysis of Deutetrabenazine and Valbenazine as VMAT2 Inhibitors for Tardive Dyskinesia: A Systematic Review

**DOI:** 10.5334/tohm.842

**Published:** 2024-03-13

**Authors:** Mohadese Golsorkhi, Jessa Koch, Farzin Pedouim, Karen Frei, Niloofar Bondariyan, Khashayar Dashtipour

**Affiliations:** 1 Loma Linda University School of Medicine, Loma Linda, California, US; 2 Pharmacy Practice, Loma Linda University School of Pharmacy, Loma Linda, California, US; 3 Division of Movement Disorders, Department of Neurology, Loma Linda University School of Medicine, Loma Linda, California, US; 4 Department of Clinical Pharmacy, School of Pharmacy, Shiraz University of Medical Sciences, Shiraz, Iran

**Keywords:** Tardive dyskinesia, VMAT2 inhibitor, valbenazine, duetetrabenazine

## Abstract

**Background:**

Tardive Dyskinesia (TD) is a neurological disorder characterized by involuntary movements, often caused by dopamine receptor antagonists. Vesicular Monoamine Transporter 2 (VMAT2) inhibitors, such as valbenazine and deutetrabenazine, have emerged as promising therapies for TD and several clinical trials have shown their efficacy. This study aims to compare the efficacy and safety profile of VMAT2 inhibitors, focusing on a recent trial conducted in the Asian population.

**Methods:**

We reviewed the PubMed, Cochrane Library, Embase database, and clinicaltrials.gov between January 2017 and October 2023, using the keywords “tardive dyskinesia” AND (“valbenazine” [all fields] OR “ deutetrabenazine “ [all fields]) AND “clinical trial”. The reviewed articles were studied for efficacy and side effects.

**Results:**

An initial search yielded 230 articles, of which 104 were duplicates. Following the title and abstract screening, 25 additional articles were excluded. A full-text review resulted in the exclusion of 96 more articles. Ultimately, four double-blind clinical trials met the inclusion criteria. The deutetrabenazine studies demonstrated significant improvements in Abnormal Involuntary Movement Scale (AIMS) scores compared to placebo, with no difference in adverse events. The valbenazine studies showed favorable results in reducing TD symptoms and were well-tolerated.

**Discussion:**

The studies reviewed in this analysis underscore the potential of deutetrabenazine and valbenazine as valuable treatment options for TD in diverse populations. Both medications demonstrated significant improvements in AIMS scores, suggesting their effectiveness in managing TD symptoms. Additionally, they exhibited favorable safety profiles, with low rates of serious adverse events and no significant increase in QT prolongation, parkinsonism, suicidal ideation, or mortality.

**Conclusion:**

The studies reviewed highlight the promising efficacy and tolerability of deutetrabenazine and valbenazine as treatments for Tardive Dyskinesia, providing new hope for individuals affected by this challenging condition.

## Introduction

Tardive dyskinesia (TD) is characterized by the insidious onset of rhythmic, repetitive, stereotypic movements involving the face, mouth, and tongue, frequently extending to the trunk and limbs as a result of exposure to dopamine receptor-blocking agents (DRBA), such as antipsychotic medications and metoclopramide [1]. The exact mechanism underlying TD is not understood, but secondary upregulation and increased sensitivity of D2 dopamine receptors, also known as the dopamine supersensitivity hypothesis, may play a role in its pathophysiology [2]. However, it may not be the exclusive cause for TD, other contributors include damage to gamma amino butyric acid (GABA)-ergic neurons in the basal ganglia, [3] neurodegeneration of striatal interneurons resulting from oxidative stress due to prolonged antipsychotic use, and the development of maladaptive synaptic plasticity in the neocortex combined with abnormal basal ganglia output causing motor program miscoding [4]. The prevalence of TD for lifetime exposure is 13.1% for second-generation antipsychotics (SGAs) and 32.4% for first-generation antipsychotics (FGAs) [5]. Moreover, around 20–35% of people who have been prescribed antipsychotics for a minimum of three months encounter TD [6]. TD symptoms can significantly affect a patient’s quality of life and lead to significant physical disability in severe cases [7]. With the expanded use of SGAs for additional on-label and off-label indications, the trend for TD may continue to rise even with fewer FGA prescriptions [5]. Despite the advancements in drug development, TD remains a challenging clinical problem that requires evaluating the options of discontinuing or reducing the doses of the offending medications. However, this approach can potentially worsen the underlying psychiatric symptoms or conversely exacerbate dyskinesia [8]. Vesicular monoamine transporter 2 (VMAT2), located mainly in neurons, has a role in storing monoamines such as dopamine, serotonin, norepinephrine, and histamine within vesicles in presynaptic cleft. When VMAT2 is inhibited, the release of monoamines is prevented and leads to reduced amount of dopamine available for binding to postsynaptic receptors [9]. Dopamine plays a critical role in other neural pathways as well. Blocking dopamine in motor circuits can cause hypersensitivity of post-synaptic dopamine receptors and an increase in dopaminergic signaling, leading to abnormal movements associated with TD [10]. VMAT2 inhibitors, such as valbenazine (VBZ) and deutetrabenazine (dTBZ), are the most novel therapies for tardive dyskinesia [11,12,13,14,15] and have been approved by the Food and Drug Administration for this indication [16,17]. Here, we conducted a systematic review of recent clinical trials investigating the efficacy and safety of dTBZ and VBZ for the treatment of TD using the Abnormal Involuntary Movement Scale (AIMS) as the primary measure. Although this scale lacks a clear separation between chorea, dystonia and excludes parkinsonism and tremor, it remains the predominant instrument in clinical trials to measure antipsychotic associated hyperkinetic movements. It achieves an ideal scenario requiring accurate assessment of changes induced by both antipsychotics and treatments targeting the underlying movement disorders [18]. Previous studies on VMAT2 inhibitors have predominantly concentrated on the population living in North America and Europe. Since the Asian population accounts for more than half of the global population, this review tries to address this demographic gap and provides valuable insights into the potential variations of VMAT2 inhibitors among diverse populations.

## Methods

### Literature Search Strategy

An extensive literature search was conducted following the guidelines outlined by the Preferred Reporting Items for Systematic Reviews and Meta-Analyses (PRISMA) to identify relevant studies. We included double-blind clinical trials that used VMAT2 inhibitors to treat TD from January 2017 to October 2023. To collect data, we searched databases including PubMed, Cochrane Library, Embase, and clinicaltrials.gov. Studies published before 2017 were excluded, as a recent systematic review covered earlier studies [19]. The search strategy in PubMed involved using the keywords “tardive dyskinesia” AND (“valbenazine” [all fields] OR “deutetrabenazine” [all fields]) AND “clinical trial” with similar terms employed in other databases.

### Study Selection

During the first level of screening, the titles and abstracts of the articles were reviewed by MG and NB. The following inclusion criteria were used: (a) articles published in English or had a published English translation; (b) articles published in a peer-reviewed journal; (c) original studies in human adults (no reviews, no animal studies); (d) original studies of any design that focused on treating TD with dTBZ or VBZ; and (e) studies evaluating AIMS by movement disorder specialists. All preclinical studies, case reports, and open label studies were excluded, as well as letters, consensus reports, and editorials. The bibliographic references of systematic reviews and meta-analyses were reviewed to address any relevant studies that may have been missed during the initial search. During the second level of screening, the full text of selected articles was assessed using the same inclusion/exclusion criteria. In both screening sessions, the authors convened to resolve any disagreement or uncertainty (Table 1).

**Table 1:** Summary of studies reported Deutetrabenazine or Valbenazine for tardive dyskinesia.


FIRST AUTHOR	LOCATION	GROUPS	SAMPLE SIZE	AGE(YEARS)	MALE(%)	TD DURATION	DIAGNOSIS	DOPAMINE RECEPTOR ANTAGONISTS	FINDINGS

DEUTETRABENAZINE									

Fernandez et al. 2017 (ARM-TD)	US and Europe (46 centers)	Deutetrabenazine 12 mg/day Up to 48 mg/day	58	55.9 ± 9.8	50.0	72.6 ± 81.7 months	– Schizophrenia/schizoaffective disorder – Bipolar disorder – Depression	– Antipsychotics (Quetiapine, Risperidone, Olanzapine)	– AIMS score difference: (LS mean [SE] –3.0 [0.45] vs –1.6 [0.46], p = 0.019) – CGIC: not significant – PGIC: not significant – mCDQ-24: not significant

		Placebo group	59	53.3 ± 10.6	45.8	76.8 ± 82.1 months			

		Total	117	54.6 ± 10.3	47.9	74.7 ± 81.5 months			

Anderson et al. 2017 (AIM-TD)	US and Europe (75 centers)	Deutetrabenazine 12 mg/day	74	57.0 ± 10.0	43.0	5.5 ± 5.4 years	– Schizophrenia/schizoaffective disorder – Bipolar disorder – Depression	– Antipsychotics	– AIMS score difference: – Deutetrabenazine 36 mg/day: –1·9 (SE 0·58, 95% CI –3·09 to –0·79; p = 0·001) – Deutetrabenazine 24 mg/day: –1·8 points (0·60, –3·00 to –0·63; p = 0·003) – Deutetrabenazine 12 mg/day: –0·7 points (0·57, –1·84 to 0·42; p = 0·217) – PGIC: not significant – mCDQ-24: not significant

		Deutetrabenazine 24 mg/day	73	55.6 ± 11.3	44.0	5.0 ± 6.0 years			

		Deutetrabenazine 36 mg/day	74	58.3 ± 11.6	43.0	5.9 ± 5.3 years			

		Placebo group	72	54.6 ± 12.1	49.0	6.0 ± 5.4 years			

		Total	293	56.4 ± 11.3	45.0	5.6 ± 5.5 years			

Valbenazine									

Hauser et al. 2017 (KINECT-3)	North America (63 centers)	Valbenazine 40 mg/day	72	55.3 ± 8.5	58.3	NR	– Schizophrenia/schizoaffective disorders – Mood disorders	– Antipsychotics: (Atypical antipsychotics: Quetiapine, Risperidone, Aripiprazole, Olanzapine, Ziprasidone), Typical antipsychotics: (Haloperidol)	– AIMS score difference: 80 mg/day: –3.2 vs. –0.1 (p < 0.001) – 40 mg/day: –1.9 vs. –0.1 (p = 0.002) – CGI-TD score: not significant

		Valbenazine 80 mg/day	79	56.0 ± 10.1	49.4				

		Placebo group	76	57.0 ± 10.5	55.3				

		Total	227	56.1 ± 9.7	54.2				

Horiguchi et al. 2022 (J-KINECT)	Japan (100 sites)	Valbenazine 40 mg/day	83	58.5 ± 14.0	45.8	NR	– Schizophrenia/schizoaffective disorder – Bipolar disorder – Major depressive disorder	– Antipsychotics	– AIMS score difference: LS mean (95% CI): – 80 mg/day: –3.6 (–4.5, –2.6, P < 0.001) – 40 mg/day: –2.2 (–3.0, –1.3, P < 0.001) – CGI-TD score difference: LS mean (95% CI) – 80 mg/day: –0.6 (–1.0, –0.3, P < 0.001) – 40 mg/day: –0.4 (–0.7, –0.1, P = 0.021)

		Valbenazine 80 mg/day	82	57.9 ± 13.6	61.0				

		Placebo group	84	60.0 ± 13.4	42.9				


AIMS: Abnormal Involuntary Movement Scale, CGIC: Clinical Global Impression of Change, CI: confidence interval, CGI-TD: Clinical Global Impression of Change–Tardive Dyskinesia, LS: least square, mCDQ-24: modified Craniocervical Dystonia Questionnaire, NR: not reported, PGIC: Patient Global Impression of Change, SE: standard error, TD: tardive dyskinesia.

### Data Extraction and Bias Assessment

Following the screening process, randomized double-blinded placebo control trials that met the predetermined inclusion criteria were chosen for systematic data extraction and quality evaluation. Each study was assessed for the possibility of bias using Cochrane criteria focusing on several types of bias such as selection, performance, attrition, detection, and reporting.

## Results

### Search Results and Characteristics

**Figure 1:**
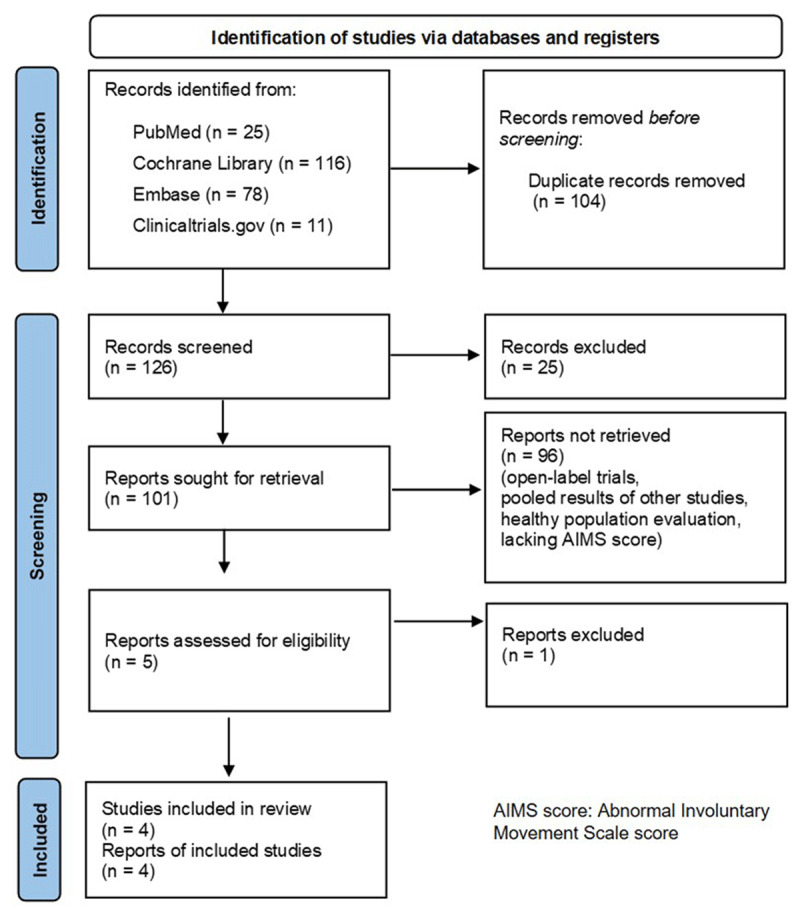
Flow diagram of current study.

Among 230 articles found in the literature search, 104 were duplicates. Out of the remaining, 25 were excluded based on the initial screening of their titles and abstracts. After conducting a comprehensive review of the full texts, an additional 96 studies were excluded from the analysis because of being open-label trials, pooling the results of other studies, focusing on healthy populations, and lacking the AIMS score measurement. As a result, four publications were selected for further evaluation. (Figure 1)

### Deutetrabenazine

Deutetrabenazine, a tetrabenazine isotope with deuterium replacing “normal” hydrogen atom, is more resistant to metabolism than tetrabenazine and requires less frequent dosing due to its longer plasma half-life [9].

Fernandez et al. (2017) conducted a study, known as randomized controlled trial of deutetrabenazine for tardive dyskinesia (ARM-TD), to assess the efficacy and safety of dTBZ in treating TD [13]. The study employed a double-blind, placebo-controlled design across 46 sites in the United States and Europe. A total of 117 patients with moderate-to-severe TD were randomized to receive either dTBZ or placebo for 12 weeks. Participants were required to have a diagnosis of TD for a minimum of three months and receive DRBA for at least three months (or one month if they were 60 years or older). The primary objective was the changes in the AIMS at specific time points by two independent movement disorder experts blinded to the study [20]. The secondary objectives included the Clinical Global Impression of Change (CGIC), Patient Global Impression of Change (PGIC), both ranging from “very much worse” to “very much improved”, and the modified Chorea Dystonia Quality of Life Scale (mCDQ-24). DTBZ was initiated at a daily dose of 12 mg (6 mg twice daily) and then adjusted weekly by 6 mg/day. The titration process continued for a maximum of six weeks until adequate control of dyskinesia was achieved, a significant adverse event occurred, or the maximum allowable dose of 48 mg/day was reached. It was followed by a six-week maintenance phase and a one-week washout period. At the end of the titration phase, the average total dosage reached 38.8 mg/day. By week four, there was a significant difference in the improvement of AIMS score between the two groups with the treatment effect of –1.5 (95% CI –2.6 to –0.4, *p = 0.007*). They concluded that dTBZ significantly reduced AIMS scores from baseline to week 12 compared to placebo (least-squares [LS] mean [standard error (SE)]: –3.0 [0.45] vs. –1.6 [0.46], *p = 0.019*; treatment difference –1.4 [0.60], 95% confidence interval [CI] – 2.6 to – 0.2). Although dTBZ group showed higher treatment success rates on the CGIC (48.2% vs. 40.4%), PGIC (42.9% vs. 29.8%), mCDQ-24 compared to placebo, these differences were not statistically significant.

Patients in both groups experienced similar frequencies of adverse events (AEs). The most common AEs in the dTBZ group vs. placebo included somnolence (13.8% vs. 10.2%), fatigue (6.9% vs. 8.5%), insomnia (6.9% vs. 1.7%), and headache (5.2% vs. 10.2%). Other symptoms like diarrhea, akathisia, anxiety, dizziness, dry mouth, upper respiratory tract infection, rash, and vomiting were also reported in both groups. Serious AEs were reported in three patients receiving dTBZ (pneumonia, manic episode, exacerbation of schizophrenia), and five receiving placebo (heroin overdose, jaw fracture, jaw infection, pneumonia, and laryngeal hypertrophy). None of which were attributed to the treatment drug.

It is noteworthy that tetrabenazine carries an FDA boxed warning for its increased risk of depression and suicidal thoughts [21]. However, the incidence of depression and suicidal ideation in dTBZ was not reported to be higher compared to placebo in this study. Yet, patients taking both dTBZ and antipsychotics showed a higher rate of psychiatric AEs compared to placebo (19.6% vs. 9.8%). Overall, the study provided strong evidence that dTBZ is a beneficial and safe treatment option for patients with established TD.

Anderson et al. conducted another study called “Addressing Involuntary Movements in Tardive Dyskinesia (AIM-TD)” at 75 locations in the US and Europe [12]. A total of 293 patients diagnosed with TD were assigned to receive one of three fixed-dose daily regimens of dTBZ (12 mg, 24 mg, or 36 mg) or placebo for 12 weeks. Patients were started on dTBZ 12 mg/day (orally, twice per day). Subsequently, they increased the dosage over four weeks until the randomized dose was achieved and sustained for eight weeks. The primary endpoint was the changes in AIMS score from baseline to week 12, and the secondary endpoints were CGIC, PGIC, and mCDQ24.

By the second week, patients in both 24 mg/day and 36 mg/day showed improvement in response to treatment (*P = 0.006* and P = *0.032*, respectively). They reported that AIMS score significantly improved in all dTBZ groups compared to the placebo from baseline to week 12. The LS mean AIMS score improved by –3.3 points (SE = 0.42) in the 36 mg/day group, –3.2 points (SE = 0.45) in the 24 mg/day group, and –2.1 points (SE = 0.42) in the 12 mg/day group. The treatment difference was –1.9 points (SE 0.58, *p = 0.001*), –1.8 points (0.60, *p = 0.003*), and – 0.7 points (0.57, *p = 0.217*) respectively vs. –1.4 points (0.41) in the placebo. Patients receiving dTBZ at doses of 24 and 36 mg/day had higher chances of achieving treatment success on the CGIC score than placebo (*P = 0.002* and *P = 0.011*, respectively). DTBZ also showed a slightly better response on PGIC and mCDQ24 scores; but the difference was not statistically significant.

Adverse events were consistent among all four groups with the exception of nervous system AEs (somnolence and headache), which was higher in dTBZ 36 mg/day (22%) compared to placebo (14%). Serious AEs, such as worsening suicidal thoughts, were reported in 5% of patients receiving dTBZ, mainly unrelated to the medication, and in 4% of patients in the control group. Notably, there was one instance of worsening suicidal thoughts on the second day of taking dTBZ 24 mg, which was possibly attributed to the offending medication. The incidence of other psychiatric AEs, such as depression, was similar in all four groups.

The authors concluded that a higher proportion of patients receiving dTBZ 24 or 36 mg/day achieved a 50% or greater improvement in their AIMS score during eight weeks, which confirms the medication’s efficacy.

### Valbenazine

In 2017, the FDA approved VBZ for the treatment of TD in adults [22]. VBZ is the valine ester of (+) α-HTBZ (the most active metabolite of TBZ) and is highly selective for VMAT2. It is a prodrug consisting of the purified alpha isomer responsible for specific and robust inhibition of VMAT2, leading to decreased risk of off-target receptor binding and consequent adverse effects. It has a slower breakdown rate, resulting in a longer half-life, allowing once-daily administration [23].

Hauser et al. presented results from a phase three trial, known as KINECT-3, evaluating the efficacy, safety, and tolerability of fixed doses of VBZ in adults with TD [11]. The trial took place in 63 centers across North America. A total of 205 patients were randomly assigned to receive VBZ 40 mg/day, or VBZ 80 mg/day, or placebo for six weeks. Consequently, 198 patients continued the study for 48 more weeks, entering the VBZ extension (VE) period and randomized to either VBZ 40 mg or 80 mg per day. The primary endpoint was a change in AIMS score from baseline to week six and week 52 for those receiving VBZ 80 mg/day compared to placebo. By week two, both VBZ doses displayed significant differences in AIMS score compared to the placebo (*p < 0.05*). They reported a significant decrease in AIMS score at week six (–3.2 VBZ 80 mg vs. –0.1 placebo, *p < 0.001*) and week 52 (–4.8 VBZ 80 mg vs. –3.0 VBZ 40 mg, *p < 0.001*). There were no significant differences between any of the VBZ dosage groups and the placebo in CGI-TD and PGI-TD scores at week six. However, during long-term treatment, a clinically meaningful improvement was observed with both scales.

After six weeks, treatment-related adverse events were found to be as high as 45.7% in both VBZ groups and increased to 69.2% after almost a year of treatment. The most commonly reported AEs for treatment groups (40 mg/day and 80 mg/day) were somnolence (3.9% and 5.3%), akathisia (1.3% and 3.3%), and dry mouth (1.3% and 3.3%, respectively). During the extension period, common reported AEs were headache (7.1%), urinary tract infection (6.6%), and diarrhea (5.6%). Also, three patients had to discontinue the study due to deterioration of their suicidal thoughts and behavior, but all were judged as unlikely related to the study medication [11].

An important limitation of previous studies is the exclusive recruitment of patients from North American and European populations. In 2022, Jun Horiguchi et al [14] published the findings of a trial on the Japanese population (J-KINECT). The study enrolled 259 patients and randomized them into three groups, placebo (n = 84), VBZ 40 mg (n = 83), or VBZ 80 mg (n = 82) for 6 weeks. Then, individuals who initially received a placebo were reassigned to either VBZ 40 mg or 80 mg for 42 more weeks (VE period). The primary objective was the change in AIMS score after six weeks. The LS mean change from baseline to week six was –2.2 in the VBZ 40 mg group compared to placebo, and –3.6 in the 80 mg group vs. placebo (both *p < 0.001*). Patients receiving either 40 or 80 mg/day of VBZ consistently showed decreased AIMS scores throughout the study. Those who transitioned from placebo to VBZ also experienced a significant change during the VE period. Both groups sustained improvements in AIMS scores by week 48. The LS mean CGI-TD scores at week six were 2.8 (80 mg/day group), 3.0 (40 mg/day group), and 3.4 (in placebo); demonstrating significant improvement in each VBZ group compared to the placebo (*P = 0.021* and *p < 0.001* respectively).

In the placebo-controlled (PC) period, more patients in VBZ groups (40 mg and 80 mg) experienced adverse events compared to the placebo (63.5% and 79.8% vs. 44.0%, respectively). The 80 mg VBZ group had more events leading to discontinuation or dose reduction. Patients receiving VBZ at 40 or 80 mg/day were more likely to experience drowsiness (11.8% and 25%, respectively) and excessive salivation (3.5% and 10.7%, respectively) during the PC period. In the extension period, common AEs reported in VBZ groups included nasopharyngitis, somnolence, hypersalivation, and insomnia. During the extended duration of treatment, two patients experienced a deterioration in their conditions of schizophrenia and depression; however, the overall results showed that patients’ psychiatric conditions remained stable during treatment with VMAT2 inhibitors.

## Discussion

Before the discovery of VMAT2 inhibitors, managing TD primarily focused on reducing the dose of offending medication, switching to alternative medications with lower risks of TD, or discontinuing the causative medication if clinically feasible. Additionally, medications such as benzodiazepines, or amantadine were sometimes employed to help alleviate TD symptoms despite their limited efficacy [24]. The introduction of VMAT2 inhibitors demonstrated a shift in addressing TD symptoms, offering a more targeted approach by modulating the dopamine system in the brain. While TBZ has shown efficacy in managing TD, its short half-life requires multiple daily doses, and its broad range of side effects restricts its widespread use. For instance, it can induce dose-dependent QT prolongation, sometimes leading to cardiac arrhythmias. DTBZ and VBZ offer significant pharmacokinetic advantages over TBZ, including longer half-life, better tolerability, and fewer side effects [9].

### Basic Characteristics

The prevalence of TD exhibits regional variations among psychiatric patients receiving antipsychotic medications, with reported rates ranging from 17% in the Asian population to 23% in Europeans and 31% in the US [5]. African-Americans have been identified as having a greater susceptibility to developing TD compared to the other racial groups [25]. The clinical trials reviewed here enrolled participants with comparable ages. Schizophrenia/schizoaffective disorders were the prominent diagnoses among patients, and atypical antipsychotics were the most prescribed medications. TD durations varied among participants, with an average of six years for dTBZ and over two years in VBZ studies [11,12,13,14].

### AIMS Score

In the AIM-TD study by Anderson et al., all tested TBZ doses significantly improved AIMS scores, with the highest dose (36 mg/day) resulting in the most substantial improvement. Patients showed a response to treatment as early as the second week, and a higher percentage of individuals in the dTBZ groups experienced at least a 50% improvement in AIMS score compared to placebo [12]. The ARM-TD study on dTBZ also demonstrated significant reductions in AIMS scores from baseline to week 12, with the most significant reductions observed in the highest dose group [13]. This study indicated early improvements in AIMS score as early as week four.Comparative analysis of VBZ’s effect in Asian and American populations showed consistent improvements in AIMS score after six weeks of treatment. The J-KINECT study reported LS mean changes of –2.3 and –3.7 in the AIMS score at week six for the 40 mg and 80 mg groups, respectively [14]. Similarly, the KINECT-3 study [11] demonstrated AIMS score change in valbenazine 40 mg and 80 mg with –1.9 and –3.2 in week six, respectively. While the magnitude of improvements varied slightly, both populations experienced positive responses to VBZ treatment. The J-KINECT study showed sustained improvements in TD symptoms over 48 weeks, with mean changes in AIMS score of –3.7 and –5.7 in the 40 and 80 mg groups, respectively. In the KINECT-3 study, changes of –3.0 and –4.8 were observed at week 48. These findings suggest the potential for VBZ to provide lasting benefits in managing TD symptoms [11,14].

### CGIC and PGIC Score

The AIM-TD study showed significant differences at week 12 on the CGIC between dTBZ 24 and 36 mg and the placebo group. Patient-reported outcome scales, PGIC, and mCDQ-24, also indicated a numerically better response in the dTBZ groups, although statistical significance was not achieved [12]. In the ARM-TD study, more patients achieved treatment success on the CGIC and PGIC with dTBZ compared to placebo; however, these differences were not statistically significant. Similarly, dTBZ-treated patients showed a greater reduction in the mCDQ-24 score from baseline to week 12 compared to placebo, but the difference was not statistically significant [13]. These findings suggest a trend towards improved treatment outcomes with dTBZ, although the lack of statistical significance emphasizes the need for larger studies to validate these observations further.

In the KINECT3 study, there were no significant differences in the CGI-TD score at week six between either dosage of VBZ and placebo. However, supportive analyses in the per-protocol population showed significant differences in favor of VBZ [11]. Conversely, in the J-KINECT study, both the 40 mg and 80 mg VBZ groups demonstrated statistically significant improvements in the CGI-TD score at week six compared to the placebo. Notably, the CGI-TD scores continued to decrease further by the end of the treatment period and returned to baseline levels during the post-treatment observation period [14]. While the findings from the KINECT3 study were less conclusive, the results from the J-KINECT study support the effectiveness of VBZ in improving TD symptoms. Further research is warranted to explore the reasons for the discrepancies between the two studies and to evaluate the long-term efficacy and safety of VBZ for TD management. The improvement in AIMS score did not completely correspond to the CGIC and PGIC outcomes. This could be attributed to differences in symptom recognition between the clinician and the patient.

### Adverse Events

**Figure 2:**
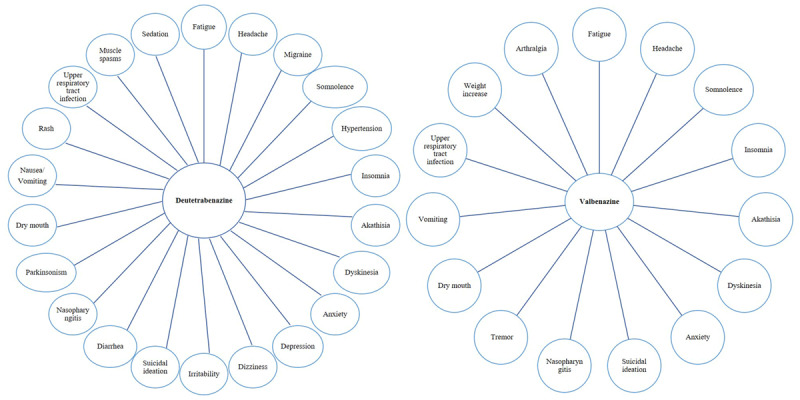
Common side effects reported with Deutetrabenazine or Valbenazine consumption.

Both dTBZ and VBZ demonstrated favorable tolerability among the majority of patients (Table 2). Data analysis showed that TRAEs ranged from 19% to 48.3% in dTBZ studies and 45.7% to 71.6% in VBZ studies. Serious AEs were reported in only 5% of medication users. Additionally, one out of ten VMAT2 inhibitor users required a dose reduction or discontinuation during treatment. The most common AEs for dTBZ were headache (5%), somnolence (4%), diarrhea (4%), fatigue (3.5%), anxiety (3.5%), and nasopharyngitis (3.2%). The rate of nervous system-related AEs was higher in the group receiving 36 mg/day of dTBZ compared to the 12 and 24 mg/day groups. Both studies reported low rates of psychiatric AEs with no observed dose-response relationship. The occurrence of side effects was comparable between VBZ and placebo, with the most frequently reported being somnolence (12%), akathisia (4%), nasopharyngitis (3.1%), and insomnia (3.1%). During the long-term treatment phase, the VBZ group reported AEs with a frequency of ≥10%, including somnolence, tremor, insomnia, worsening of schizophrenia, nasopharyngitis, and hypersalivation. (Figure 2) Continued monitoring and evaluation of adverse events are necessary to ensure patient safety in clinical practice.

**Table 2:** Summary of Adverse Events Reported in Duetetrabenazine and Valbenazine Studies.


	POPULATION	AE (%)	SERIOUS AE (%)	AE LED TO DOSE REDUCTION (%)	HEADACHE (%)	SOMNOLENCE (%)	NASOPHARYNGITIS (%)	ANXIETY (%)	DEPRESSION (%)	DIARRHEA (%)	SUICIDAL IDEATION (%)	NAUSEA/VOMITING (%)	FATIGUE (%)	DRY MOUTH (%)	INSOMNIA (%)	AKATHISIA (%)	DYSKINESIA (%)	DEATHS (%)

Deutetrabenazine																		

Fernandez et al.	Deutetrabenazine	70.7	5.2	10.3	5.2	13.8	NR	3.4%	0.0	5.2	0.0	1.7	6.9	3.4	6.9	5.2	NR	0.0

	Placebo	61.0	8.5	5.1	10.2	10.2	NR	6.8	1.7	5.1	1.7	5.1	8.5	10.2	1.7	0.0	NR	0.0

	Total	65.8	6.8	7.7	7.7	12.0	NR	5.1	0.8	5.1	0.8	3.4	7.7	6.8	NR	2.6	NR	0.0

Anderson et al.	Deutetrabenazine 12 mg/day	49.0	3.0	0.0	7.0	0.0	5.0	4.0	1.0	1.0	0.0	1.0	1.0	4.0	NR	0.0	0.0	0.0

	Deutetrabenazine 24 mg/day	44.0	8.0	1.0	3.0	1.0	4.0	3.0	4.0	4.0	3.0	1.0	3.0	0.0	NR	1.0	1.0	1.0

	Deutetrabenazine 36 mg/day	51.0	5.0	4.0	7.0	4.0	3.0	4.0	1.0	7.0	1.0	1.0	4.0	3.0	NR	0.0	1.0	1.0

	Placebo	47.0	6.0	0.0	6.0	4.0	1.0	3.0	0.0	3.0	0.0	10.0	1.0	0.0	NR	0.0	0.0	0.0

	Total	48.0	6.0	1.0	5.0	2.0	3.0	3.0	2.0	4.0	1.0	3.0	2.0	2.0	NR	<1	1.0	1.0

Valbenazine																		

Hauser et al.	Valbenazine 40 mg/day	40.3	5.6	NR	2.8	5.6	NR	1.4	NR	NR	4.2	0.0	2.8	6.9	1.4	4.2	0.0	0.0

	Valbenazine 80 mg/day	50.6	7.6	NR	2.5	5.1	NR	2.5	NR	NR	1.3	3.8	1.3	0.0	2.5	2.5	3.8	1.3

	Placebo	43.4	3.9	NR	2.6	3.9	NR	0.0	NR	NR	5.3	0.0	1.3	1.3	1.3	1.3	0.0	0.0

	Total	45.0	5.7	NR	2.6	4.8	NR	1.3	NR	NR	3.5	1.3	1.8	2.6	1.8	2.6	1.3	0.4

Horiguchi et al.	Valbenazine 40 mg/day	63.5	NR	NR	1.2	11.8	7.1	1.2	1.2	NR	NR	NR	NR	NR	2.4	4.7	NR	NR

	Valbenazine 80 mg/day	79.8	NR	NR	4.8	25.0	4.8	4.8	3.6	NR	NR	NR	NR	NR	6.0	6.0	NR	NR

	Placebo	44.0	NR	NR	2.4	2.4	7.1	0.0	1.2	NR	NR	NR	NR	NR	1.2	1.2	NR	NR

	Total	62.4	NR	NR	2.8	13.0	6.3	2.0	2.0	NR	NR	NR	NR	NR	3.2	4.0	NR	NR


AE: Adverse events.

The J-KINECT study revealed higher incidences of TEAEs for VBZ compared to KINECT-3. While the exact reason for the relatively higher incidence of TEAEs in the J-KINECT study is not yet clear, it is worth noting that there were no significant differences in the occurrence of severe TEAEs between this study and KINECT-3. It is plausible that the incidence of such events may increase with higher VBZ exposure. The patients’ background factors that may account for the differences between the Japanese and non-Japanese populations remain unknown and warrant further investigation.

### QT Prolongation

In the ARM-TD study, no significant differences were observed regarding QTc interval prolongation between the treatment groups (p > 0.153). Similarly, in the AIM-TD study, among patients with a normal baseline QTc interval, more individuals in the placebo group (13%) experienced abnormal values (>450 ms) compared to the dTBZ groups with different doses (ranging from 4% to 10%). While VBZ may prolong the QT interval, the degree of prolongation is not considered clinically significant at recommended doses. However, caution is advised, and VMAT2 inhibitors should be avoided in patients with congenital long QT syndrome or those with arrhythmias associated with prolonged QT intervals. These findings underscore the importance of careful cardiac monitoring in patients receiving these medications, with pre-existing QT interval abnormalities or conditions predisposing them to prolonged QT intervals.

### Suicidal Ideation and Attempt

The use of DRBA in psychiatric conditions is linked to an elevated suicidality risk [26]. TBZ carries an FDA-boxed warning regarding suicidality; [27] However, in a retrospective study including more than 500 patients diagnosed with HD using TBZ, participants showed a relatively low risk of suicidality with or without a history of depression compared to the control group (OR = 0.61, p = 0.043 and OR = 0.57, p = 0.028, respectively) [28].

In ARM-TD, there were no reports of worsening suicidal ideation/behavior with dTBZ. In AIM-TD, three patients (1%) reported suicidal ideation, with two cases unrelated to the treatment and one patient in the dTBZ 24 mg/day group had potentially treatment-related suicidal ideation. In another study by Jankovic et al., long-term effects of dTBZ use in 228 children and adolescents diagnosed with tics showed no increase in depression or suicidality measures with prolonged treatment [29].

The KINECT-3 study showed increased suicidal thoughts in both placebo and treatment groups. With four in the placebo group, two in the 80 mg/day group, and three in the 40 mg/day group. Therefore, no significant difference was observed between the VBZ and the placebo. There was one suicide attempt, which the investigator determined was unlikely to be associated with the study medication. However, monitoring suicidal ideation in patients with TD who are is necessary.

### Mortality

Mortality in clinical trials is an essential consideration in evaluating the safety of investigational drugs. Two patients in the dTBZ group died, but none of them were related to the study medication. In the Japanese population, seven patients passed away, with no apparent drug link in six cases. In the 40-mg VBZ group, one death had an undetermined cause potentially linked to the study medication, but the investigators noted the patient’s use of 20 other medications as a possible contributor.

### Parkinsonism

Previously parkinsonism has been reported in 15% of patients taking TBZ [30]. In real-world reports, instances have been documented where the use of VMAT2 inhibitors resulted in drug-induced parkinsonism (DIP) [31,32]. Per the prescribing information, the frequency of parkinsonism for VBZ is 3%, and in the AIM-TD study, only one patient in the 36 mg dTBZ group experienced parkinsonism, leading to a dose reduction. In ARM-TD, slight improvements in parkinsonism were seen in both dTBZ and placebo groups over 12 weeks.

The absence of significant parkinsonism suggests these drugs may be a favorable option for patients with TD, but clinicians should be cautious and maintain a high index of clinical suspicion for DIP, irrespective of the dose.

### Patient Access and the Latest Market Innovation

It is important to acknowledge the critical consideration of patient access and the economic implications of every new medication. Market access plays a pivotal role in determining the practical impact and sustainability of treatment strategies. In a recent study, claims database (from 2001–2019) was reviewed to extract data on medication names, number of prescription days supplied, and associated costs in the US. The average monthly expenses for VMAT2 inhibitors to treat TD were $6409, $5424, and $1747, for VBZ, DTZ, and TBZ respectively. Notably, the median (25^th^ percentile, 75^th^ percentile) out-of-pocket expenses for a 30-day supply exhibited variability, with VBZ priced at $32 ($1-$422), DTZ at $35 ($1-$365), and TBZ at $29 ($1-$193) [33]. Unfortunately, for individuals with TD, limited treatment options exist. In many cases where we cannot reduce the DRBA dosage, VMAT2 inhibitors stand as their sole recourse for managing disease symptoms. Often, patients must navigate the complexities of insurance coverage and seek assistance from patient support organizations such as the National Organization for Rare Disorders (NORD) to bridge the financial gap and ensure a consistent supply of their vital medications. Therefore, the cost-effectiveness and affordability of the medication are pivotal factors that warrant further exploration to ensure widespread availability and benefit to the patient population.

In February 2023, the FDA approved dTBZ extended release (XR), a once-daily formulation that offers a simplified dosing regimen compared to the twice-daily administration required for the original formulation [34]. This XR formulation can be taken with or without food, offering consistent plasma levels by minimizing plasma fluctuations. The XR tablet has an immediate-release outer coating and an extended-release osmotic core, with approximately 27.5% of the medication being promptly released from the outer coating. The tablet core is hydrated inside the body as water permeates through the semi-permeable membrane. The remaining 72.5% of the medication is released at a consistent rate through the orifice, maintained by a constant osmotic pressure difference. The AEs associated with the XR formulation are expected to be similar to those observed with the twice-daily formulation.

In September 2023, the FDA accepted the new drug application for VBZ oral granule sprinkle capsules formulation, setting an action date of April 2024 [35]. It is intended to be sprinkled on soft food for those patients who have difficulty swallowing or prefer not to take the whole capsule. Additionally, the introduction of VBZ 60 mg to the market provides an optimized therapeutic option for many individuals suffering from TD, highlighting advancements in addressing patients in this field.

## Conclusion

In conclusion, both dTBZ and VBZ have shown promising results in treating TD with favorable improvements in AIMS scores. These medications offer advantages over previous treatments, including longer half-lives and potentially better tolerability. While they have demonstrated safety profiles and minimal risks of suicidal ideation or mortality in clinical trials, careful monitoring of side effects and cardiac parameters remains essential in clinical practice. Further research and larger-scale studies are needed to confirm their long-term effectiveness and safety, especially in diverse patient populations.

## References

[1] Frei K, Truong DD, Fahn S, Jankovic J, Hauser RA. The nosology of tardive syndromes. Journal of the neurological sciences. 2018; 389: 10–6. DOI: 10.1016/j.jns.2018.02.008 29433810

[2] Frei K. Tardive dyskinesia: Who gets it and why. Parkinsonism & Related Disorders. 2019; 59: 151–4. DOI: 10.1016/j.parkreldis.2018.11.017 30522959

[3] Cadet JL, Lohr JB. Possible Involvement of Free Radicals in Neuroleptic-Induced Movement Disorders Evidence from Treatment of Tardive Dyskinesia with Vitamin E. Annals of the New York Academy of Sciences. 1989; 570(1): 176–85. DOI: 10.1111/j.1749-6632.1989.tb14918.x 2576510

[4] Teo JT, Edwards MJ, Bhatia K. Tardive dyskinesia is caused by maladaptive synaptic plasticity: a hypothesis. Movement Disorders. 2012; 27(10): 1205–15. DOI: 10.1002/mds.25107 22865512

[5] Carbon M, Hsieh C-H, Kane JM, Correll CU. Tardive dyskinesia prevalence in the period of second-generation antipsychotic use: a meta-analysis. The Journal of clinical psychiatry. 2017; 78(3): 20738. DOI: 10.4088/JCP.16r10832 28146614

[6] Ward KM, Citrome L. Antipsychotic-related movement disorders: Drug-induced parkinsonism vs. tardive dyskinesia—Key differences in pathophysiology and clinical management. Neurology and therapy. 2018; 7: 233–48. DOI: 10.1007/s40120-018-0105-0 30027457 PMC6283785

[7] McEvoy J, Gandhi SK, Rizio AA, Maher S, Kosinski M, Bjorner JB, et al. Effect of tardive dyskinesia on quality of life in patients with bipolar disorder, major depressive disorder, and schizophrenia. Quality of Life Research. 2019; 28: 3303–12. DOI: 10.1007/s11136-019-02269-8 31435866 PMC6863950

[8] Waln O, Jankovic J. An update on tardive dyskinesia: from phenomenology to treatment. Tremor and other hyperkinetic movements. 2013; 3. DOI: 10.5334/tohm.165 PMC3709416 23858394

[9] Koch J, Shi W-X, Dashtipour K. VMAT2 inhibitors for the treatment of hyperkinetic movement disorders. Pharmacology & Therapeutics. 2020; 212: 107580. DOI: 10.1016/j.pharmthera.2020.107580 32454050

[10] Peckham AM, Nicewonder JA. VMAT2 Inhibitors for Tardive Dyskinesia—Practice Implications. Journal of Pharmacy Practice. 2019; 32(4): 450–7. DOI: 10.1177/0897190018756512 29455579

[11] Hauser RA, Factor SA, Marder SR, Knesevich MA, Ramirez PM, Jimenez R, et al. KINECT 3: a phase 3 randomized, double-blind, placebo-controlled trial of valbenazine for tardive dyskinesia. American Journal of Psychiatry. 2017; 174(5): 476–84. DOI: 10.1176/appi.ajp.2017.16091037 28320223

[12] Anderson KE, Stamler D, Davis MD, Factor SA, Hauser RA, Isojärvi J, et al. Deutetrabenazine for treatment of involuntary movements in patients with tardive dyskinesia (AIM-TD): a double-blind, randomised, placebo-controlled, phase 3 trial. The Lancet Psychiatry. 2017; 4(8): 595–604. DOI: 10.1016/S2215-0366(17)30236-5 28668671

[13] Fernandez HH, Factor SA, Hauser RA, Jimenez-Shahed J, Ondo WG, Jarskog LF, et al. Randomized controlled trial of deutetrabenazine for tardive dyskinesia: the ARM-TD study. Neurology. 2017; 88(21): 2003–10. DOI: 10.1212/WNL.0000000000003960 28446646 PMC5440239

[14] Horiguchi J, Watanabe K, Kondo K, Iwatake A, Sakamoto H, Susuta Y, et al. Efficacy and safety of valbenazine in Japanese patients with tardive dyskinesia: A multicenter, randomized, double-blind, placebo-controlled study (J-KINECT). Psychiatry and Clinical Neurosciences. 2022; 76(11): 560–9. DOI: 10.1111/pcn.13455 36114799 PMC9826124

[15] Chen S, Zhang XJ, Xie WJ, Qiu HY, Liu H, Le WD. A New VMAT-2 inhibitor NBI-641449 in the treatment of Huntington disease. CNS Neuroscience & Therapeutics. 2015; 21(8): 662–71. DOI: 10.1111/cns.12425 26122704 PMC6495663

[16] Uhlyar S, Rey JA. Valbenazine (Ingrezza): The first FDA-approved treatment for tardive dyskinesia. Pharmacy and Therapeutics. 2018; 43(6): 328. 29896031 PMC5969209

[17] Park B. Austedo XR, a Once-Daily Formulation of Deutetrabenazine, Gets FDA Approval. MPR Monthly Prescribing Reference. 2023; NA–NA.

[18] Martino D, Karnik V, Bhidayasiri R, Hall DA, Hauser RA, Macerollo A, et al. Scales for Antipsychotic-Associated Movement Disorders: Systematic Review, Critique, and Recommendations. Movement Disorders; 2023. DOI: 10.1002/mds.29392 37081740

[19] Khorassani F, Luther K, Talreja O. Valbenazine and deutetrabenazine: vesicular monoamine transporter 2 inhibitors for tardive dyskinesia. American Journal of Health-System Pharmacy. 2020; 77(3): 167–74. DOI: 10.1093/ajhp/zxz299 31974564

[20] Stacy M, Sajatovic M, Kane JM, Cutler AJ, Liang GS, O’Brien CF, et al. Abnormal involuntary movement scale in tardive dyskinesia: Minimal clinically important difference. Movement Disorders. 2019; 34(8): 1203–9. DOI: 10.1002/mds.27769 31234240 PMC6772010

[21] Shen V, Clarence-Smith K, Hunter C, Jankovic J. Safety and efficacy of tetrabenazine and use of concomitant medications during long-term, open-label treatment of chorea associated with Huntington’s and other diseases. Tremor and Other Hyperkinetic Movements. 2013; 3. DOI: 10.5334/tohm.129 PMC3822048 24255799

[22] Gupta H, Moity AR, Jumonville A, Kaufman S, Edinoff AN, Kaye AD. Valbenazine for the Treatment of Adults with Tardive Dyskinesia. Health Psychology Research. 2021; 9(1). DOI: 10.52965/001c.24929 PMC8801818 35106396

[23] O’brien CF. Treatment of hyperkinetic movement disorders. Google Patents; 2017.

[24] Egan MF, Apud J, Wyatt RJ. Treatment of tardive dyskinesia. Schizophrenia Bulletin. 1997; 23(4): 583–609. DOI: 10.1093/schbul/23.4.583 9365997

[25] Bhidayasiri R, Boonyawairoj S. Spectrum of tardive syndromes: clinical recognition and management. Postgraduate medical journal. 2011; 87(1024): 132–41. DOI: 10.1136/pgmj.2010.103234 21131613

[26] Schwartz S, Dinkla L, Pullen J, Bernard R, Kumar A. Characteristics of Inpatients Prescribed Dopamine Receptor Blocking Agents. Psychopharmacology Bulletin. 2021; 51(4): 51. 34887599 10.64719/pb.4418 PMC8601763

[27] Yero T, Rey JA. Tetrabenazine (Xenazine), an FDA-approved treatment option for Huntington’s disease–related chorea. Pharmacy and Therapeutics. 2008; 33(12): 690. 19750050 PMC2730806

[28] Schultz JL, Killoran A, Nopoulos PC, Chabal CC, Moser DJ, Kamholz JA. Evaluating depression and suicidality in tetrabenazine users with Huntington disease. Neurology. 2018; 91(3): e202–e7. DOI: 10.1212/WNL.0000000000005817 29925548

[29] Jankovic J, Coffey B, Claassen DO, Jimenez-Shahed J, Gertz BJ, Garofalo EA, et al. Safety and Efficacy of Long-Term Deutetrabenazine Use in Children and Adolescents with Tics Associated with Tourette Syndrome: An Open-Label Extension Study. Movement Disorders Clinical Practice. 2023; 10(9): 1388–98. DOI: 10.1002/mdc3.13849 37772282 PMC10525047

[30] Kenney C, Hunter C, Jankovic J. Long-term tolerability of tetrabenazine in the treatment of hyperkinetic movement disorders. Movement disorders: official journal of the Movement Disorder Society. 2007; 22(2): 193–7. DOI: 10.1002/mds.21222 17133512

[31] Vasireddy RP, Sokola B, Guduru Z. New generation VMAT2 inhibitors induced parkinsonism. Clinical Parkinsonism & Related Disorders. 2020; 3. DOI: 10.1016/j.prdoa.2020.100078 PMC8298827 34316656

[32] Akbar U, Kim DS, Friedman JH. Valbenazine-induced parkinsonism. Parkinsonism & Related Disorders. 2020; 70: 13–4. DOI: 10.1016/j.parkreldis.2019.11.021 31785443

[33] Reynolds EL, Gallagher G, Hill CE, Banerjee M, Mante A, Esper GJ, et al. Costs and utilization of new-to-market neurologic medications. Neurology. 2023; 100(9): e884–e98. DOI: 10.1212/WNL.0000000000201627 36450601 PMC9990429

[34] AUSTEDO® XR (deutetrabenazine) extended-release tablets, for oral use 2023/02 [updated 02/2023]. Available from: https://www.accessdata.fda.gov/drugsatfda_docs/label/2017/208082s000lbl.pdf.

[35] Neurocrine Biosciences Announces U.S. FDA Accepts New Drug Application for INGREZZA® (valbenazine) Oral Granules Sprinkle Formulation 2023 [Available from: https://neurocrine.gcs-web.com/news-releases/news-release-details/neurocrine-biosciences-announces-us-fda-accepts-new-drug].

